# Preliminary study on mechanical characteristics of maxillofacial soft and hard tissues for virtual surgery

**DOI:** 10.1007/s11548-020-02257-1

**Published:** 2020-10-31

**Authors:** Yu Zhuang, Jie Chen, Qingcheng Liu, Fan Zou, Yuheng Lin, Qinglong An, Hongbo Yu

**Affiliations:** 1 Department of Oral and Cranio-maxillofacial Surgery, Shanghai Ninth People’s Hospital, College of Stomatology, Shanghai Jiao Tong University School of Medicine, No. 639 Zhizaoju Road, Huangpu District, Shanghai, 200011 China; 2 Shanghai Key Laboratory of Stomatology and Shanghai Research Institute of Stomatology, Shanghai, 200011 China; 3 National Clinical Research Center for Oral Diseases, Shanghai, 200011 China; 4 State Key Laboratory of Mechanical System and Vibration, School of Mechanical Engineering, Shanghai Jiao Tong University, No. 800 Dongchuan Road, Minhang District, Shanghai, China

**Keywords:** Maxillofacial soft and hard tissues, Insertion and cutting, Elasticity modulus, Hardness, Haptic feedback, Virtual surgery

## Abstract

**Purpose:**

Virtual surgery system can provide us a realistic and immersive training environment, in which haptic force-feedback gives operators ‘touching feeling.’ Appropriate deformation models of soft and hard tissues are required for the achievement of real-time haptic feedback. To improve accuracy of modeling and haptic feedback simulation for maxillofacial virtual surgery, mechanical characteristics of soft and hard tissues should be explored.

**Methods:**

Craniofacial soft tissues from one male and female cadavers were divided into two layers: skin and muscle. Maxillofacial tissues were divided into frontal, chin, temporalis, masseter regions. Insertion and cutting process were conducted using VMX42 5-axis linkage system and recorded by piezoelectric dynamometer. Maximum stiffness values were analyzed, and insertion curves before puncture were fitted using a polynomial model. Elasticity modulus and hardness of maxillofacial hard tissues were measured and analyzed using Berkovich nanoindentation.

**Results:**

Tissues in different maxillofacial regions, as well as from different layers (skin and muscle), displayed various mechanical performance. Maximum stiffness values and cutting force of soft tissues in male and female had significant difference. The third-order polynomial was demonstrated to fit the insertion curves well before puncture. Furthermore, elasticity modulus and hardness of enamel were significantly greater than that of zygoma, maxilla and mandible.

**Conclusion:**

Mechanical properties of hard tissues are relatively stable, which can be applied in virtual surgery system for physical model construction. Insertion model and cutting force for soft tissues are meaningful and applicable and can be utilized to promote the accuracy of response for haptic feedback sensations.

## Introduction

Surgical training is a long procedure, which consists of knowledge acquisition and experience obtain, gained by assisting or performing surgical procedures on patients under appropriate supervision. Technical ability is one of the most important parts of competence in surgery. Unskilled operation and insufficient experience can lead to medical errors. Among of them, tactile force perception is vital in surgical performance. However, this “feeling” required in surgery can only be obtained through long-term practice and training [1].

Fortunately, virtual surgery system can provide us a realistic and immersive training environment, in which haptic force-feedback gives operators ‘touching feeling,’ and real-time image feedback makes the experience more vivid [2, 3]. Surgeons can improve their operation skills, shorten clinical training time and reduce medical accidents by virtual system.

According to the “three-stage” theory proposed by Satava [4], now the development of virtual surgical system is at stage Two, in which the mechanical properties of different tissues are studied for the construction of appropriate physical models. Constructing soft and hard tissue deformation models based on real mechanical properties is vital in virtual surgical simulation system. However, soft tissues have characteristics of anisotropy, non-uniform viscoelasticity and time-dependent deformation. The boundary deformation of soft tissues is complicated, which makes it difficult to construct accurate deformation model in mathematical and physical views.

To achieve real-time deformation and haptic feedback, appropriate deformation models of soft and hard tissues are required. Multiple strategies, including finite element (FE), boundary element (BE) and mass-spring model (MSD), can be utilized to construct deformation models based on physical characteristics of tissues [5, 6]. Moreover, mass-spring/damper model (MSDM) method has been utilized to accomplish soft and hard tissue deformation simulation and haptic feedback in maxillofacial modeling [7]. While the virtual surgery fidelity is still unsatisfactory for the reason of lacking biomechanical parameters of real tissues [8].

Previously, the mechanical parameters of mandibular bone from cadaver, including elastic modulus, intensity and sawing force were measured. Based on this, the biomechanical model with image and hepatic feedback by means of boundary element method was established and the osteotomy, fixation and prediction of orthognathic surgery were realized in realistic and immersive virtual reality environment [9–11]. However, the mechanical properties of other maxillofacial bones like maxilla, zygoma have not been measured. At the same time, lacking deformation and hepatic feedback of soft tissues hinder the final effects of the integral surgery simulation.

To establish the real-time deformation model with VR-based image and haptic feedback for virtual simulation, biomechanical properties of hard tissues were measured and real insertion and cutting force for soft tissues were recorded and modeled for haptic feedback.

## Materials and methods

### Material preparation

**Fig. 1:**
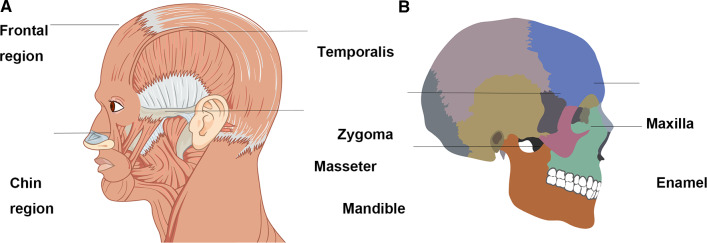
Subregions of soft and hard tissues in maxillofacial region

Craniofacial hard and soft tissues were harvested from one male and female donated cadavers killed in car accident, aged 40 and 30 years old, respectively, and samples were stored in frozen condition. To precisely describe mechanical properties of soft tissues in different maxillofacial areas, they were divided into two layers: one layer consisting of epidermis, dermis and subcutaneous tissue, the other including muscle tissue. In view of wide application of intraoral mucosal incision in orthognathic surgery, mucosa tissues were also separated and measured. Skin tissues in frontal, chin and masseter regions, and temporalis and masseter tissues were dissected (Fig. 1a). Hard tissues from different regions, including zygoma, maxilla, mandible and dental enamel, were measured for elasticity modulus and hardness (Fig. 1b). Before the measurement, sample tissues were thawed, dissected using knife or saw and temporarily soaked within saline in ice box. The interval time between harvesting and measurement were within twelve hours.

### Equipment setup and the detection of insertion and cutting force

**Fig. 2:**
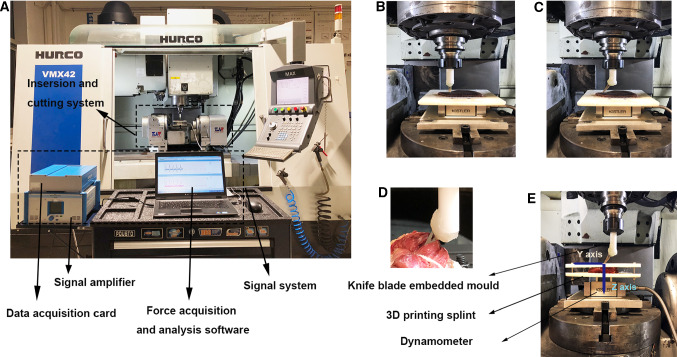
The detection system. **a** Experimental setup, including signal system (data acquisition card, signal amplifier, force acquisition and analysis software) and insertion and cutting system. **b** Insertion process, the blades perpendicular to tissue. **c** Cutting process, the blades displaying 45 degree. **d** Distinct deformation of soft tissues during cutting process without fixation. **e** Cutting system, with 3D printing splint fixing tissues

The detections were conducted using VMX42 5-axis linkage system (HURCO, USA) to provide power and stable velocity. Insertion and cutting force were measured by piezoelectric dynamometer (Kistler 9256C2, Switzerland), and the acquisition frequency was set to 4 × 10^3^ Hz. 3D printing splint was designed to fix the soft tissues during insertion and cutting process. The detection system is shown in Fig. 2a. All specimens were soaked in physiological saline before measurement.

The surgical knife blade was embedded into a customized 3D printing mold and immobilized in machine by a clamp (Fig. 2e). To simulate the real cutting process, the blade first punctured the tissue (Fig. 2b), which was called insertion process below, and after reaching bone surface the blade performed 45 degree cutting (Fig. 2c), which was called cutting process below. Soft tissues were mounted on the 3-orientation dynamometer for insertion and cutting force measurement (Fig. 2e). The measuring system was composed of the Kistler piezoelectric dynamometer, data acquisition card, signaling amplifier and computer controller. For the reason that soft tissues are flexible, it was difficult to fix them during cutting process (Fig. 2d); thus, 3D printing splint was designed and used to immobilize them effectively (Fig. 2e).

The tissue insertion speeds were set at 0.5, 1, 1.5, 2 mm/s, and the cutting speeds were set at 0.5, 1, 1.5 mm/s, according to actual operational process in surgery. The experiments were repeated five times at different speeds. Considering the directionality of muscle fibers, muscle cutting force was measured perpendicular and parallel to muscle fibers separately. The speeds and depths in insertion and cutting process could be controlled and adjusted by VMX42 5-axis system, to obtain experimental data under different conditions with variable speeds or depths.

Considering effects of different genders and areas on mechanical characteristics, soft tissues from male and female donors in different maxillofacial areas were measured, respectively. Cutting force could be obtained in both horizontal (*Y* axis) and vertical dimensions (*Z* axis) using four-component piezoelectric dynamometer. During insertion process, velocity, time and insertion force were recorded for data analysis. Insertion curve (displacement–force) fitting was performed using Matlab software (MathWorks, USA).

To further describe mechanical behavior in insertion process of soft tissues. Matlab software was utilized to reconstruct insertion model to match real insertion process before puncture, based on displacement–force data measured during experiments. Polynomial model

1 $$ Y = aX^{3} + bX^{2} + cX + d $$

was used as fitting curves, in which *y* (N) represented insertion force, *x* (mm) represented displacement, and *a*, *b*, *c*, *d* were coefficients.

### Berkovich nanoindentation

The specimens included cortical bone samples of zygoma, maxilla, mandible and dental enamel, which were immersed in 0.9% saline immediately after being removed from cadavers. The hard tissues were embedded in PMMA (polymethyl methacrylate) resin in silastic mold, after rinsed and cleaned with 0.9% physiological saline. The hard tissues were embedded in columnar resin after polymerization, with a size of 5 mm × 5 mm × 2 mm and polished up with the roughness of surface being 92 nm utilizing diamond grind disks for rough polish and polish fluids for fine polish.

**Fig. 3:**
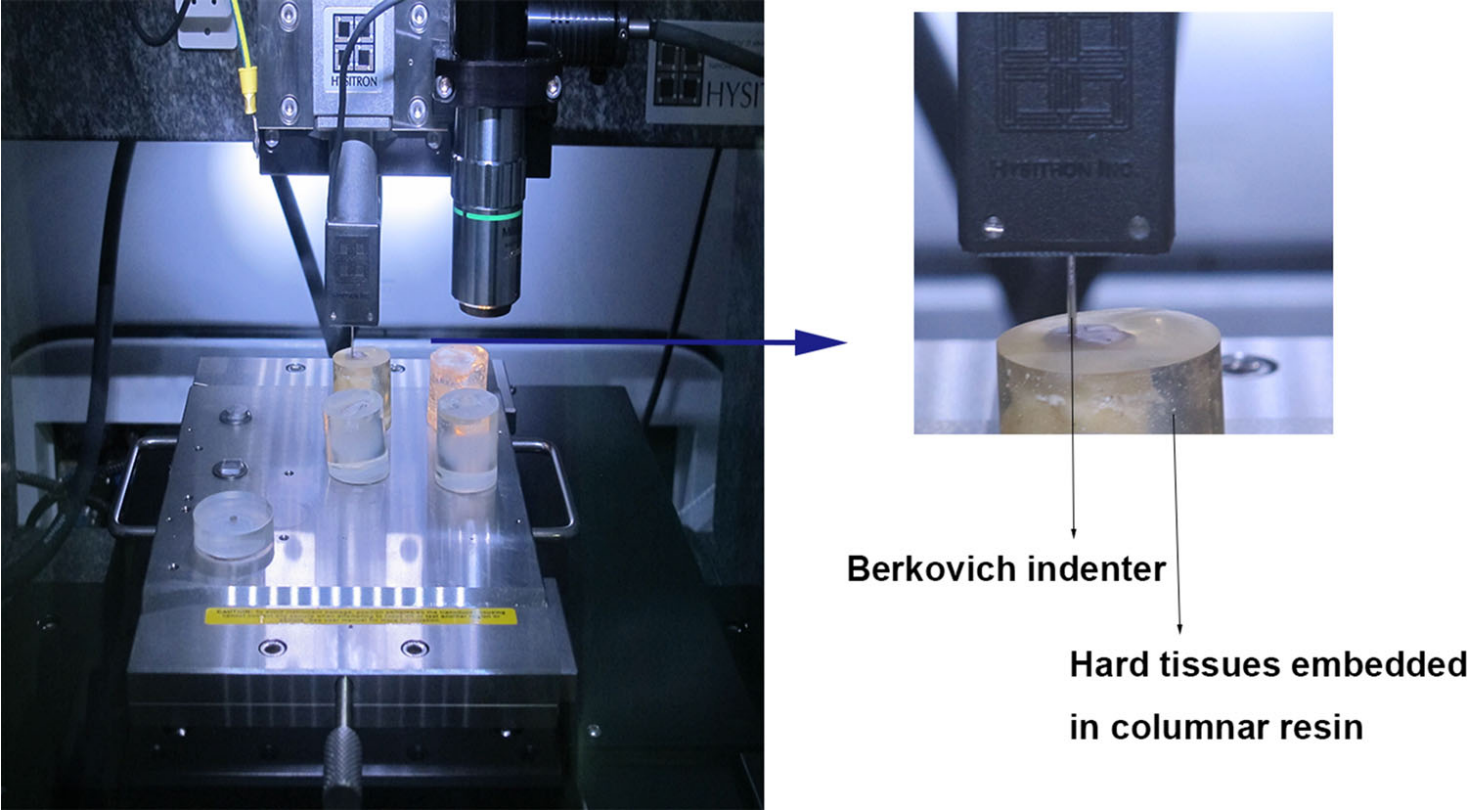
The equipment setup for Berkovich nanoindentation

Berkovich nanoindentation instrument TI 950 TriboIndenter (Hysitron, Inc., Minneapolis, MN) was utilized to measure elasticity modulus and contact hardness of maxillofacial hard tissues. The equipment setup is shown in Fig. 3. The Berkovich indenter (triangular pyramid) performed displacement in the *Z* direction, and specimens were allowed for movement in the *X*, *Y* direction. The thermal drift rate of every indentation was corrected to make the limitation below 0.05 nm/s. Every indentation consisted of a linear loading process, a keeping part (2 s) set at 10,000 μN maximum loading, and an unloading process. Six indentations were made in each sample, and the spacing distance was set as 10 μm [12].

Force–displacement curves obtained in the experiments were analyzed to compute the elasticity modulus and contact hardness, according to the theory of Oliver and Pharr [13]. The contact hardness is defined as:

2 $$ H_{\text{c}} = \frac{{F_{{\rm max}} }}{A} $$

in which $H_{\text{c}}$ represents contact hardness, $F_{{\rm max}}$ represents maximum loading, and $A$ represents contact area.

The relationship for curves is represented by:

3 $$ S = \frac{2}{\sqrt \pi }E_{\text{r}} \sqrt A $$

in which *S* represents contact stiffness, the slope of initial unloading point, and *A* represents contact area.$E_{\text{r}}$, the reduced modulus, is determined by:

4 $$ \frac{1}{{E_{\text{r}} }} = \frac{{1 - v_{\text{s}}^{2} }}{{E_{\text{s}} }} + \frac{{1 - v_{\text{i}}^{2} }}{{E_{\text{i}} }} $$

where $E_{\text{s}}$, $v_{\text{s}}$ are, respectively, Young’s modulus and Poisson ratio of specimens, and $E_{\text{i}}$, $v_{\text{i}}$ represent the same parameters of indenter. The rationality of this equation is dependent on the conception that the material is isotropic.

### Statistical analysis

The data for insertion and cutting force were analyzed using independent-samples *t* test, and for elasticity modulus and contact hardness utilizing one-way analysis of variance (ANOVA), and SNK *q* analysis by SPSS 22.0 software (Chicago, IL, USA). *p* < 0.05 was considered to be of statistically difference. Data were displayed as mean ± standard deviation (SD).

## Results

### Acquisition of insertion force for soft tissues

**Fig. 4:**
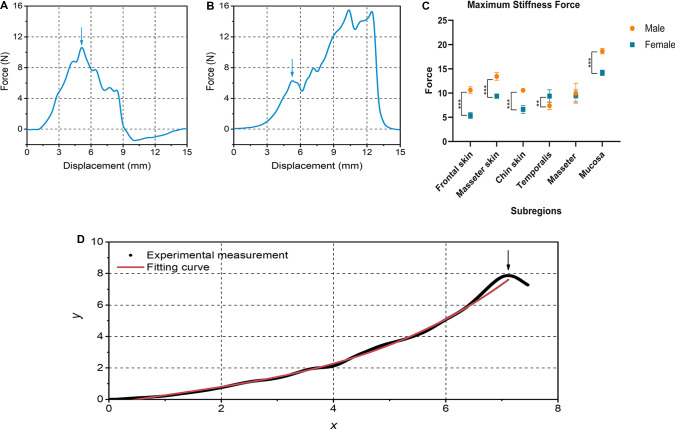
Insertion process. (a-b) Displacement–force curves for insertion process: **a** chin skin; **b** temporalis, the arrow showed the maximum stiffness force. (The arrows refer to the maximum stiffness force). **c** Maximum stiffness force of soft tissues in different maxillofacial regions. **p < 0.01; ***p < 0.001. **d** Polynomial fitting curve for insertion process before puncture. The black line was real displacement–force curve, and the blue line was fitting curve

The insertion process was recorded using piezoelectric dynamometer, and data were output to computer for further analysis. Time–force curves were obtained, and displacement–force were subsequently calculated based on constant velocity. Displacement–force curves, which are displayed on Fig. 4a, b, showed that two main phases were involved in insertion process: pre-puncture and post-puncture. Puncture events happened at the point, designated as a peak, a sudden force drop, following a stable force raise. In Fig. 4a, reason for force in reverse direction was that blade first moved down and then moved up to continue next insertion process. The maximum stiffness force at the turning point is shown in Table 1. Maximum stiffness force measured at different velocities had no significant difference.

**Table 1:** Maximum stiffness force for soft tissues in different maxillofacial regions

	Frontal skin (N)	Masseter skin (N)	Chin skin (N)	Temporalis (N)	Masseter (N)	Mucosa (N)
Male	10.621 ± 0.672	13.443 ± 0.789	10.578 ± 0.187	7.377 ± 0.795	9.959 ± 2.045	18.664 ± 0.560
Female	5.356 ± 0.611	9.367 ± 0.459	6.633 ± 0.800	9.393 ± 1.293	9.417 ± 1.237	14.192 ± 0.551

Mechanical characteristics of soft tissues in insertion process were examined, and the results (Fig. 4c) showed that the maximum stiffness values of skin and mucosa in male were significantly greater than those in female. While the maximum stiffness value of male temporalis was smaller than that of female. The maximum stiffness value of mucosa was greater than that of skins and muscles.

Matlab software was utilized to reconstruct an insertion model to match real insertion process before puncture. Based on polynomial model, the fitting curve for insertion process before puncture was established. The fitting curve matched the real displacement–force curve well, and value of R-square was 0.999 (Fig. 4d).

### Acquisition of cutting force for soft tissues

**Fig. 5:**
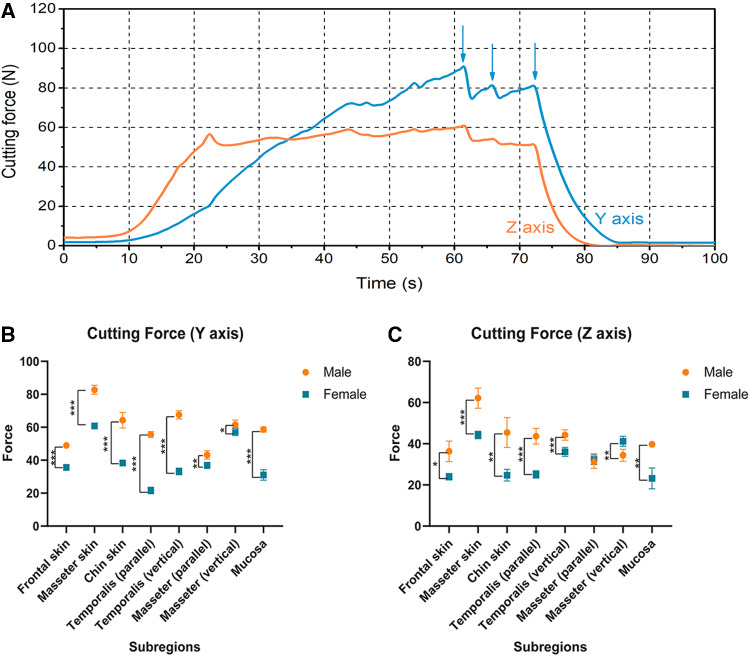
Cutting process. **a** Time–force curves during cutting process. Blue line represented force in *Y* dimension, and orange line represented force in *Z* axis. The arrows marked the points at which muscle fibers were cut off. **b**, **c** Cutting force, including *Y* axis and *Z* axis force, for soft tissues in different maxillofacial regions. *p < 0.05; **p < 0.01; ***p < 0.001

Force in three dimensions (*X*, *Y* and *Z*) was recorded during cutting process. Time–force curves are shown in Fig. 5a. During horizontal cutting process, loading force was composed of vertical force in *Z* axis, and horizontal force in *Y* axis. Horizontal force in *X* axis was so tiny that it can be ignored, thus it was not analyzed here. Muscle fibers were cut off during cutting process, which made some peaks in *Y* axis curve. During the horizontal cutting process, force firstly increased time-dependently, after reaching the point at which muscle fibers began to be cut off, cutting force in *Y* axis tended to fluctuate in a limited range. Force in *Z* axis increased time-dependently in 90° downward cutting process and then maintained stable at some value in horizontal cutting process.

Cutting force in *Y* and *Z* axis for soft tissues in different maxillofacial regions is shown in Table 2. Cutting forces at different velocities had no significant difference.

**Table 2:** Cutting force for soft tissues in different maxillofacial regions

		Frontal skin (N)	Masseter skin (N)	Chin skin (N)	Temporalis-parallel (N)	Temporalis-vertical (N)	Masseter-parallel (N)	Masseter-vertical (N)	Mucosa (N)
*Y* axis	Male	48.963 ± 1.479	82.729 ± 2.726	64.258 ± 4.716	55.621 ± 1.749	67.531 ± 2.478	43.204 ± 2.482	61.604 ± 2.769	58.641 ± 1.578
	Female	35.664 ± 0.865	60.775 ± 1.208	38.258 ± 1.693	21.613 ± 1.693	33.165 ± 2.046	36.815 ± 1.859	57.376 ± 2.453	31.041 ± 3.164
*Z* axis	Male	36.297 ± 5.023	62.145 ± 4.921	45.449 ± 7.252	43.624 ± 3.802	44.242 ± 2.522	31.204 ± 3.202	34.408 ± 2.879	39.692 ± 1.312
	Female	23.984 ± 1.605	44.264 ± 1.810	24.704 ± 2.831	25.093 ± 1.793	36.084 ± 2.163	32.467 ± 2.587	41.184 ± 2.451	23.161 ± 5.091

Cutting force in *Y* axis for male was significantly greater than that for female. Force in Z axis for male was also greater than that for female in significance, except for masseter cutting. Only force in masseter (vertical to muscle fiber) for female was significantly greater than that for male (Fig. 5b, c).

### Measurement of elasticity modulus and contact hardness of hard tissues

**Fig. 6:**
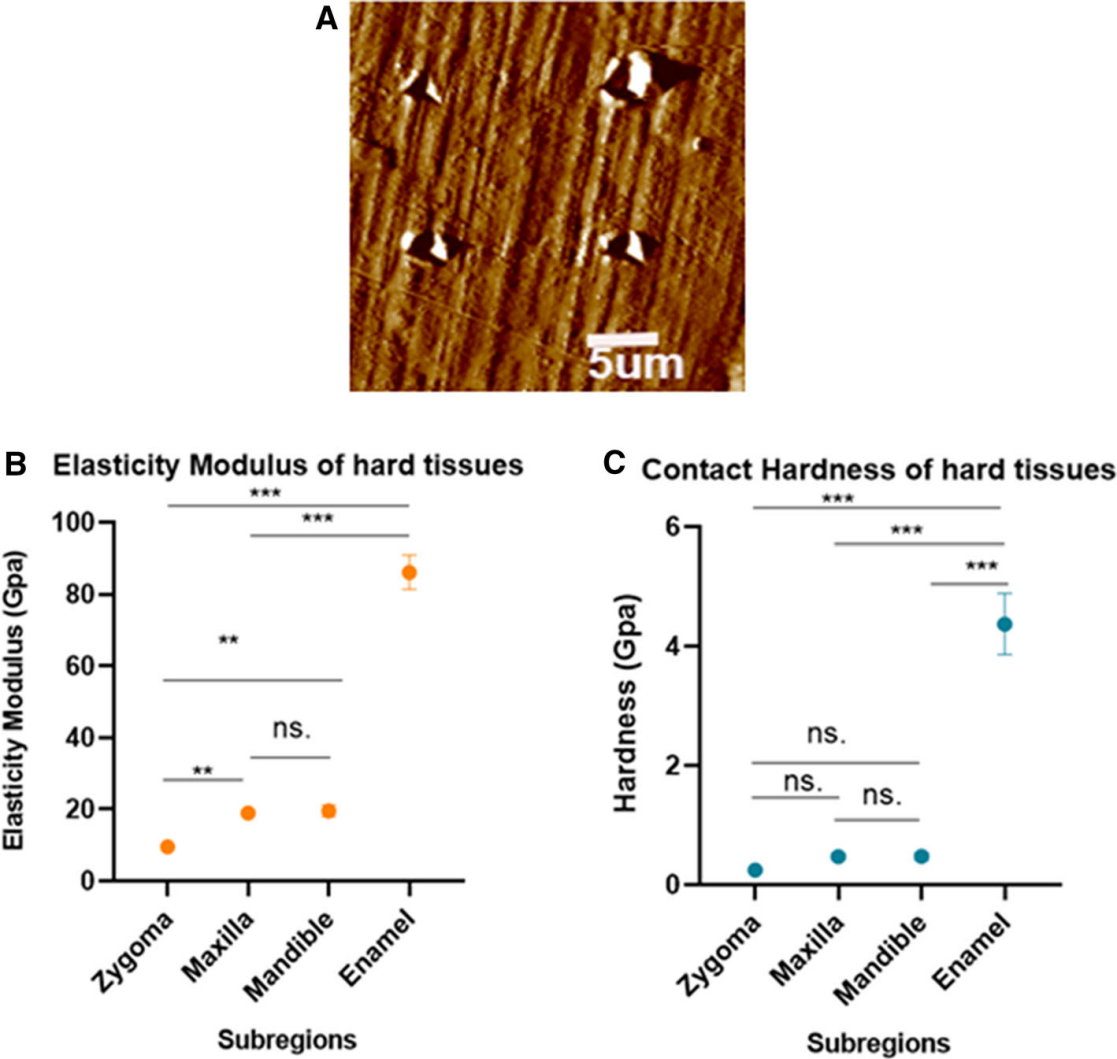
Berkovich nanoindentation. **a** The micrographs for indentation tests. Topography scanning after indentation, showing marks of the indentations. **b**, **c** Summary data of elasticity modulus and contact hardness of hard tissues. **p < 0.01; ***p < 0.001, *ns* no significance

The indentations, shown in Fig. 6a, were approximately 800 nm in depth. The summary data of elasticity modulus (*E*) and contact hardness (*H*) of hard tissues (zygoma, maxilla, mandible and enamel) are shown in Table 3 and Fig. 6b, c. All data were presented in the form of average over indentations in all specimens. The results indicated that values of *E* and *H* of enamel were remarkably greater than that of maxillofacial bones. Values of *E* for maxilla and mandible were distinctly greater than that of zygoma. But there was no significant difference between jaw and zygoma in values of *H*. Values of *E* and *H* of maxilla and mandible had no significant difference.

**Table 3:** Elasticity modulus and contact hardness of hard tissues

	Zygoma	Maxilla	Mandible	Enamel
Elasticity modulus (Gpa)	9.447 ± 0.178	18.992 ± 0.681	19.564 ± 1.627	86.159 ± 4.789
Contact hardness (Gpa)	0.242 ± 0.069	0.474 ± 0.048	0.476 ± 0.062	4.374 ± 0.513

## Discussion

### Recording insertion curves, obtaining maximum stiffness force and modeling insertion force

**Fig. 7:**
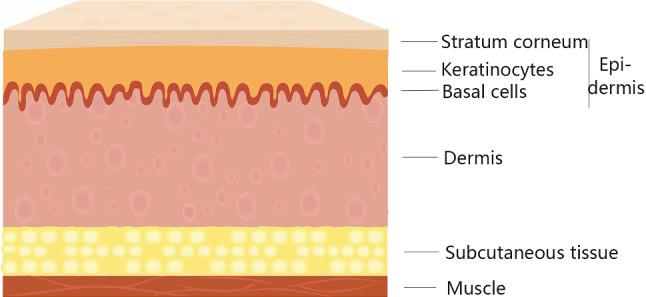
Schematic illustration of skin and muscle structure

The force–displacement curves showed the change of force increasing and sharp force drop, at which point the tissue layer was punctured. Several puncture events happened in insertion process, but there were some differences in the meaning of puncture event happening in skin and muscle tissues. Different anatomic structures accounted for the differences. Structure of skin and muscle is shown in Fig. 7. Skin is consisted of epidermis and dermis. Epidermis is stratified squamous epithelium, while the dermis is dense connective tissue with elastic and collagen fibers, which make it elastic and tough. Dermis is thicker than epidermis and has abundant sweat glands, hair follicles, blood vessels, nerves and, etc. In Fig. 4a, epidermis was firstly punctured, then dermis, and subsequent puncture events happened because of multiple structures like blood vessels, nerves, and, etc., in dermis. Muscle tissue is consisted of muscle fibers, which are filamentous. Many muscle fibers form a muscle bundle, and large muscle bundles are finally combined into a whole muscle. Fascia, wrapped around each muscle, penetrates between muscle groups and forms a muscle interval. In Fig. 4b, muscles bundles could trigger puncture events, and fascia insertion could cause sharper and higher peaks than bundles.

Characteristic structures of skin and muscle also account for the different mechanical performance in male and female specimens. The thickness of keratinized cells in epidermis, and the strength of elastic fibers and collagen fibers in dermis are variable in different regions and genders. Stratum corneum in male is generally thicker than that in female, and muscle fibers of male are stronger than fibers of female, which might account for the larger stiffness forces in male tissues.

The effects of stiffness, static and dynamic friction can be integrated and utilized to update the resistance in virtual simulation. Three essential forces form the consequent force, including stiffness force, friction force and cutting force, shown as:

5 $$ f_{\text{insertion}} \left( x \right) = f_{\text{stiffness}} \left( x \right) + f_{\text{friction}} \left( x \right) + f_{\text{cutting}} \left( x \right) $$

Stiffness force forms pre-puncture force, and friction and cutting force form post-puncture force [14].

**Fig. 8:**
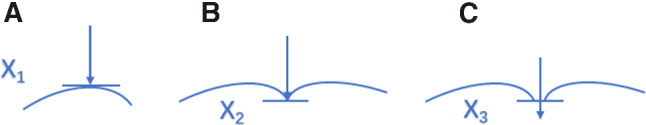
Schematic diagram for locations of knife tip at different periods. **a** Before puncture; **b** puncture; **c** after puncture

Elasticity of soft tissues accounts for stiffness force in insertion process. According to Simone et al., the relationship between stiffness force and displacement of knife tip is described as following:

6 $$ f_{\text{stiffness}} = \left\{ {\begin{array}{*{20}l} 0 \hfill & {\quad x < x_{1} } \hfill \\ {f\left( x \right)} \hfill & {\quad x_{1} \le x \le x_{2} } \hfill \\ 0 \hfill & {\quad x > x_{3} } \hfill \\ \end{array} } \right. $$

in which *x* represents displacement of knife tip. Locations of *x*_1_, *x*_2_, *x*_3_ are displayed in Fig. 8, and *f*(*x*) represents one-dimensional stiffness model. Stiffness force increases displacement-dependently, till force reaches the maximum stiffness level (Fig. 8b, $x_{1} \le x \le x_{2}$). After tissue punctured, stiffness force is reduced to zero, and further insertion force is derived from friction and cutting force (Fig. 8c, $x > x_{3}$).

Stiffness force model *f*(*x*) is established to simulate the insertion process more precisely and reveals the connection between knife depth and counter-acting mechanical force before puncture. Nonlinear strategies are used for modeling because of inevitable deformation of soft tissues before puncture. One nonlinear equation, demonstrated by d’Aulignac et al. in human thigh modeling, was used in our experiments, and the relation is shown as:

7 $$ f\left( x \right) = \frac{x}{ax + b} $$

where *x* represents insertion depth, and coefficients *a* and *b* were matched to fit the deformation curves. While smaller *R*-square values were obtained using the third-order polynomial model (Eq. 1). The polynomial model matches the insertion process well, which makes it more applicable in further virtual surgical simulation.

The simulated models fit the real curves well, although it is impossible to construct a model that matches the real data perfectly. There are still some problems. One is that abrasion and deflection occurred in knife blade during insertion and cutting process, and we assume that the tip remains unchangeable in this study. Furthermore, interior structures including blood vessels, nerves and fascia, which can trigger additive puncture peaks, are not involved in this model. Our data for modeling are obtained by in vitro experiments, and it is difficult to acquire in vivo data.

The displacement–force curves can also be applied in insertion simulations during multiple epidural anesthesia. The average insertion force values, measured in needle insertion process for epidural anesthesia, were approximately 6.037 N (Newtons) and 1.974 N, respectively, in skin and fat tissues [15]. Porcine skin was widely applied in measurement of biomechanical parameters on account of its easy availability. Vaughan et al. [16] utilized porcine models to estimate the characteristics of tissues in vitro for haptic feedback application. And they further integrated the tissue stiffness results from porcine models into VR medical training simulators [17].

Some attempts were also made to construct force models for further virtual insertion process simulation [18]. A study on force–displacement model of epidural insertion in Delft University of Technology showed that the simulated line was consistent with real curve till reaching ligamentum flavum (force maintaining in 5 N) and then increased to 16 N linearly, with a sharp decline to 9 N when reaching epidural space subsequently.

Soft tissues tend to have higher viscoelasticity and bio-extensibility, and the mechanical response is also related to the frequency and loading time, which make it more difficult to determine mechanical properties of soft tissues.

### Recording cutting curves and obtaining cutting force

Cutting process was recorded to acquire cutting force. The primary difficulty we met in cutting process was that soft tissues especially muscles were difficult to fix because of their flexibility (Fig. 2d), which made it difficult to cut off muscle fibers. To settle this problem, 3D printing splints were firstly designed and utilized in this study, which effectively fixed the tissues. Different velocities, including 0.5, 1 and 1.5 mm/s, were attempted in cutting experiments, and no significant difference between different velocities was found. The resulting cutting force is composed of friction force and cutting force. Cutting force is indispensable to slice the tissue and considered as a constant. Friction force is generated because of tissue adhesion, occurring together with the knife blade.

Muscle tissues are composed of directional muscle fibers; thus, it is necessary to cut the muscles in parallel and vertical directions, respectively, to get precise cutting force. Muscles are composed of abundant muscle bundles. When muscle bundles are cut off in cutting process, peaks are formed in curves (Fig. 5a). Fascia in muscle tissue is tougher than muscle bundles, and a sharper peak would be formed when cutting.

### Elastic properties of biological tissues measured by nanoindentation

Nanoindentation provides us an ideal strategy to determine mechanical properties of viscoelastic and anisotropic materials, like biological tissues [19, 20]. There have been researches showing that elastic characteristic of bone at microscopic level may be different from that at macroscopic level [21]. Since bone, with hierarchical structure, has microstructural characteristics, nanoindentation serves as an ideal modality to measure intrinsic mechanical characteristics at tissue level (under millimeter scale) [22]. The obtained force–displacement curves at nanometer scale make it accessible to indentation modulus and elastic characteristic.

Nanoindentation was utilized to measure elasticity modulus and contact hardness of hard tissues (zygoma, maxilla, mandible and enamel) in this study. Generally hard tissues such as bones and teeth have higher elastic modulus and hardness than soft tissues. The average elasticity modulus and hardness of dental enamel, *E* = 86.159 Gpa, *H* = 4.374 Gpa, are distinctly greater than that of maxillofacial bones. Dental enamel is considered to be the hardest structure in organism because of the highest mineralization level. The percentage of hydroxyapatite composition in enamel is 96%, and that in bone is approximately 65% [23], which accounts for the highest *H* and *E* values in dental enamel.

Several researches also studied the mechanical properties of multiple bones using indentation. Cortical and trabecular bone might possess different elastic characteristics. Rho et al. [21] found that the average elasticity modulus of vertebral trabeculae was 13.4 GPa, and that of cortex was 22.4 GPa. Zysset et al. [24] found that elasticity modulus of femur cortex was 20.1 GPa, and of trabecular bone was 11.4 Gpa. Rho et al. [25] also found that modulus levels measured in different directions were discrepant, which might also account for the difference between modulus measured at microscopic and macroscopic levels. Mechanical properties of bones from different regions within one individual ranged widely [24], and further studies are required to explore mechanical characteristics of biological tissues.

There are still limitations in our preliminary experiments on mechanical properties of biological tissues. One male and female cadaver samples were measured, and the measuring environment was not completely consistent with physiological condition. In our further study, to acquire more convincing results, adequate samples are required, and the sample could be soaked in saline during measuring process to simulate physiological environment.

## Conclusion and further perspectives

Haptic feedback is indispensable for surgeon training in virtual maxillofacial surgery, in which constructing appropriate physical models is the vital problem to be solved. To improve modeling accuracy, insertion and cutting process of soft tissues were recorded, and mechanical properties of hard tissues were measured. Tissues in different maxillofacial regions, as well as from different layers (skin and muscle) displayed various mechanical performance. Maximum stiffness values and cutting force of some tissues in male and female had significant difference. The third-order polynomial was demonstrated to fit the insertion curves well before puncture. Furthermore, the elasticity modulus and contact hardness of maxillofacial bones (including zygoma, maxilla, mandible and enamel) were significantly different.

The data can be applied in virtual surgery system for model construction. Appropriate force models can be used to promote tactile feedback sensations, to imitate the resisting force on knife blade better during insertion process of soft tissue. Mechanical properties of hard tissues and cutting force of soft tissues can be utilized in physical modeling for the realization of ideal haptic feedback.
